# Too hot for comfort: Elevated temperatures influence gene expression and exceed thermal tolerance of bigmouth shiners, *Ericymba dorsalis*

**DOI:** 10.1111/jfb.70268

**Published:** 2025-11-05

**Authors:** Ella K. Humphrey, Jonathan J. Spurgeon, Lizbeth Bowen, Robert E. Wilson, Shannon C. Waters‐Dynes, Braxton M. Newkirk, Sarah A. Sonsthagen

**Affiliations:** ^1^ School of Natural Resources University of Nebraska‐Lincoln Lincoln Nebraska USA; ^2^ U.S. Geological Survey, Nebraska Cooperative Fish and Wildlife Research Unit, School of Natural Resources University of Nebraska‐Lincoln Lincoln Nebraska USA; ^3^ U.S. Geological Survey Western Ecological Research Center Davis California USA; ^4^ Nebraska State Museum University of Nebraska‐Lincoln Lincoln Nebraska USA

**Keywords:** critical thermal maximum, environmental change, heat shock proteins, phenotypic plasticity, thermal tolerance

## Abstract

Environmental and associated ecosystem change may affect the persistence of fish species based on their ability to adapt to changing conditions, including decreasing flows and rising water temperatures. Exceeding the thermal tolerances of stream fish will likely result in a loss of ability to maintain metabolic processes. We evaluated the critical thermal maximum (CTmax) of bigmouth shiner (*Ericymba dorsalis*) and analysed the expression of heat shock protein 70 messenger RNA (mRNA) (HSP70) to quantify a thermal stress response over a gradient of temperatures (25°C–31°C). *E. dorsalis* HSP70 mRNA expression was upregulated in response to temperatures >25°C, indicating a stress response. This study supports the existence of a thermal stress threshold for *E. dorsalis*. The frequency at which this threshold is exceeded may increase under forecasted future climate scenarios for Nebraska.

## 1 INTRODUCTION

Environmental change may alter the magnitude, frequency and duration of weather events, thereby altering hydrological and thermal regimes in aquatic systems (Palmer et al., 2009; Stott, 2016). Aquatic and semi‐aquatic ectotherms, including fish, are vulnerable to hydrological shifts, as their thermal requirements are directly influenced by fluctuations in water temperature and flow patterns. Therefore, unsuitable conditions may cause species to decline (Payne et al., 2021). Within lotic systems, disturbances such as droughts and floods can substantially alter thermal regimes (Palmer et al., 2009; Poff, 1997; Rieman & Isaak, 2010). For example, droughts reduce a system's connectivity, whereby isolated pools that may experience high temperatures and poor water quality are formed (Lake, 2000). Similarly, over‐appropriation of groundwater, exacerbated by drying climates, can deplete groundwater causing an alluvial aquifer to cease groundwater inputs into a stream, thereby fragmenting a system (Falke et al., 2011). Conversely, floods can displace fish, degrade water quality through excess runoff and scour ideal habitats (Lake, 2000). Assessments of aquatic biodiversity found environmental and associated ecosystem change to be a dominant force depauperating species in lotic systems (Allan & Flecker, 1993; Xenopoulos et al., 2005). Furthermore, global extinction and extirpation rates of freshwater biodiversity are comparable to or higher than rates for terrestrial biodiversity (Heino et al., 2009; Sala et al., 2000). The current risk to freshwater biodiversity exemplifies why it is important to couple hydrological and thermal stressors in future climate predictions (Segurado et al., 2016).

Riverine fish must cope with shifting thermal regimes to persist (Hopper et al., 2020). Fish species adjust their range in pursuit of temperatures that optimize physiological functions (Comte & Grenouillet, 2013). Globally, riverine fish assemblages are shifting towards species better adapted to warm‐slow flowing water, a trend likely driven by climate change and anthropogenic disturbances to hydrological regimes (Comte et al., 2021). However, small‐bodied fishes may be especially susceptible to extirpation as low dispersal capability would force them to take an adaptation‐in‐place strategy rather than tracking suitable thermal niches (Troia et al., 2019). As a result, disturbances to both hydrological and thermal regimes may lead to substantial shifts in species abundances within a pre‐existing assemblage or the extirpation of species from an area. Understanding how environmental change influences the thermal tolerances of species may help guide best management practices.

Quantifying thermal tolerances establishes a baseline for predicting how organisms may respond to shifting thermal regimes. When the upper limit of thermal tolerance is known, current and future temperatures can be interpreted in terms of potential risk. In fish, the critical thermal maximum (CTmax) estimates the upper lethal temperature threshold measured as the point at which the fish loses equilibrium in trials where fish are exposed to acute warming temperatures. Thermal tolerances can be further constrained at the molecular level by the denaturation of proteins important for metabolic functions (Pörtner, 2002). To maintain metabolic processes when temperatures exceed thermal stress thresholds, fish produce heat shock proteins (HSPs), which help mitigate some protein damage caused by rapid temperature shifts (Feder & Hofmann, 1999). Upregulation of HSPs can occur minutes to hours after exposure to heat stress (Blake et al., 1991; Healy et al., 2010; Kyprianou et al., 2010) and ensure survival under stressful conditions (Kaur et al., 2025; Penny & Pavey, 2023). Temperatures that initiate HSP upregulation indicate that the fish is experiencing thermal stress before reaching CTmax (Manzon et al., 2022). Gene expression studies quantify the amount of messenger RNA (mRNA) transcribed and later translated into functional proteins like HSPs. Investigating HSP regulation through gene expression analysis may enable the quantification of thermal stress thresholds based on differential gene expression. Therefore, we may be able to predict how fish populations respond to environmental change by studying variations in individual expression to thermal changes in their environment.

Variations in thermal tolerances may arise from intraspecific variation attributed to phenotypic plasticity, where a single genotype expresses different phenotypes in response to environmental changes (Sommer, 2020). Acclimation, a facet of phenotypic plasticity, occurs when organisms are exposed to varying temperatures over days or months. The temperature exposures can trigger changes in physiological traits and potentially shift thermal tolerances (Kingsolver & Huey, 1998). For example, Potts et al. (2021) observed that pugnose shiner (*Notropis anogenus*) individuals acclimated to the highest temperature treatment over 4 months, exhibited larger gills, presumably to aid in oxygen uptake, and demonstrated higher thermal tolerances based on CTmax. Although thermal acclimation enables organisms to persist in the short term, long‐term exposure to altered thermal regimes over several generations may drive and necessitate evolutionary change (Fangue et al., 2006). Thermal adaptations to current climatic conditions may not suffice for projected climate scenarios because the rate at which the climate is warming is predicted to exceed many organisms' adaptive capacities (Barnosky et al., 2011; Bennett et al., 2021). Conversely, species that do not currently experience temperatures close to their thermal maximum may be able to persist through adaptation and/or phenotypic plasticity (Somero, 2012). For example, adaptations in protein‐coding sequences and increased selection for specific gene variants of HSPs may occur (Logan & Cox, 2020; Somero, 2010).

Fishes within Great Plains streams, where hydrological disturbances are common, have adapted to disturbances by tolerating poorer water quality, reproducing periodically or opportunistically and dispersing to refuge habitats (Dodds et al., 2004; Fausch et al., 2002; Labbe & Fausch, 2000). The Nebraska Sandhills is a unique ecoregion within the Great Plains where a shallow depth to groundwater enables cool‐water streams (Bleed & Flowerday, 1998). Cool‐water streams in the Sandhills provide thermal refugia for threatened small‐bodied fishes and may be critical in an era of environmental change where hydrological and thermal regimes are predicted to become more extreme (Dodds et al., 2004; Hoagstrom et al., 2011; Howell et al., 2010; Meisner, 1990; Newkirk, 2024; Schneider et al., 2011; Spooner, 2023; Westhoff & Paukert, 2014). Many fish species already live close to their thermal maximum; therefore, heightened extremes in a frequently disturbed environment could outpace the ability of those species to cope with rapid changes (Comte & Olden, 2017; Tomanek, 2008; Walters et al., 2018).

To assess the potential effects of environmental change on the ability of Great Plains fishes to cope with thermal extremes, we conducted thermal tolerance experiments on bigmouth shiners (*Ericymba dorsalis*), a stream and river generalist native to the Great Plains and the Sandhills ecoregion. The objectives of this research were to (1) quantify the CTmax of *E. dorsalis* and (2) identify a thermal stress threshold temperature based on heat shock protein 70 (HSP70) mRNA expression.

## 2 METHODS

### 2.1 Fish collection and maintenance

**FIGURE 1:**
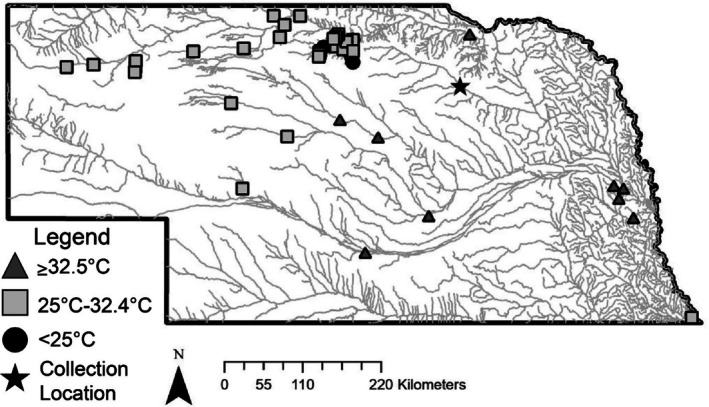
Map of Nebraska streams and rivers. Our sample site is denoted with a star. Sites (*N* = 42) with U.S. Geological Survey stream gauges or temperature loggers that exceed 25°C and 32.5°C between June and August 2015–2023 are noted. Temperature data were acquired from Nebraska Game and Parks Commission 2024 or available from USGS water data for the nation (U.S. Geological Survey, 2016).

We collected *E. dorsalis* from the South Fork of the Elkhorn River (42.251710, −98.341791) using a 6.35 mm mesh with a 4.57 m straight seine net (Figure 1). On 6 August 2023, we collected 10 *E. dorsalis* to ensure that individuals could acclimatize to captivity. We transported fish in coolers containing ice packs to avoid heat stress and bubblers to maintain dissolved oxygen levels. We measured and weighed each fish and placed them in one of the three 113‐L (30‐gal) aquaria in the rack system. To prevent disease transmission, no additional fish were introduced to this aquarium. On 17 August 2023, we collected 108 additional fish from the same location at the South Fork of the Elkhorn River. The 108 fish were quarantined in a 1135‐L (300‐gal) circular tank. We treated all 108 fish with Metroplex (Seachem Laboratories, Madison, GA, USA) from 20 to 25 August 2023 for a fungal infection that resulted in the loss of 55 individuals. Once the fungus was no longer visible, we stopped medicating with Metroplex and monitored fish health for 23 days before experiments commenced. Experiments commenced on 19 September 2023 and concluded on 6 December 2023. We divided the remaining 53 fish between the two empty 113‐L aquaria (26 and 27 fish in each tank) in the rack system. Each 113‐L aquarium contained sand substrate to promote the natural foraging behaviour of *E. dorsalis* (Hrabik, 2015; Quist et al., 2005). We fitted each aquarium with an air‐powered sponge filter and an AquaClear‐70 power filter.

We monitored the water quality and temperature of the three aquaria daily using a Freshwater Master Test Kit (API, Chalfont, PA, USA) and a YSI (Xylem Inc., Washington, DC, USA). Each aquarium's temperature depended on ambient room temperature and ranged from 16.8°C to 19.7°C. Fish were exposed to a photoperiod of 12 h of light and 12 h of darkness. We performed 50% water changes weekly to maintain water quality with tap water pretreated with activated carbon filters and ultraviolet (UV) sterilizers. We did not detect elevated levels of ammonia (<0.01 mg/L), nitrite (<0.01 mg/L) or nitrate (<40 mg/L) suggesting that our filters were sufficient to maintain water quality in the tanks given the number of fish present. Additionally, we siphoned out debris and removed algae from the glass of the tanks periodically. We fed thawed frozen brine shrimp daily and with occasional feedings of Grub Pie formula as a supplemental diet (Repashy Ventures, Oceanside, CA, USA) until satiation.

### 2.2 Critical thermal maximum experiment

We used an experimental set‐up consisting of a 45‐L cooler housing a 2‐L beaker that contained a single fish to measure CTmax. We heated the water using an 800‐W cobalt titanium aquarium heater controlled with a thermostat (BronzeOX) secured to the side of the cooler and monitored temperature using a sensor (Vernier, Beaverton, OR, USA) positioned in the 2‐L beaker. Changes in the temperature were recorded using a LabPro device (Vernier, Beaverton, OR, USA). We used a small filter to circulate water within the cooler to avoid thermal stratification. We used an external battery‐powered aerator to supply air to the beaker to keep oxygen saturation above 5 mg/L [mean = 8.35, standard deviation (SD) = 0.62]. We acclimated each fish for 20 min to the experimental set‐up before inducing thermal stress. We attempted to raise the water temperature by 0.3°C/min as in previous studies (Becker & Genoway, 1979; Morgan et al., 2018). We had a mean heating rate of 0.27°C/min (SD = 0.01). The CTmax trials were concluded once an individual lost equilibrium, defined as failing to maintain a dorso‐ventrally upright position for 10 s (O'Donnell et al., 2020).

Following CTmax trials, fish were either immediately euthanized by rapid concussion followed by pithing (*N* = 5) or were returned to their aquarium and allowed to recover overnight to monitor survival to ensure that the thermal stress event was not lethal before euthanasia the following morning (*N* = 8). All fish (*N* = 8) survived the 24‐h post‐treatment. We took lengths to the nearest 1.0 mm and weights to the nearest 0.1 g of each euthanized fish. Fulton's condition factor (K) was calculated to assess fish condition (Ricker, 1975). Final lengths and weights of CTmax experimental fish were not significantly different from the final lengths and weights of fish used in HSP experiments (weight, *p* = 0.22; length, *p* = 0.19).

### 2.3 Heat shock protein gene expression experiment

We used the same materials and similar methods from the CTmax experiment for the HSP experiment. The heating rate was set to 0.06°C/min (Table 1). We used two Cobalt Aquatics 100‐W heaters to allow for a slower heating rate (Cobalt Aquatics, Rock Hill, SC, USA). Groups of three randomly selected fish were acclimated to the experimental set‐up for 20 min in a separate aerated beaker. The groups were raised to a temperature corresponding to specific treatment groups: low (21°C–23°C; *N* = 10), moderate (25°C–27°C; *N* = 10) and high (29°C–31°C; *N* = 16). We held fish within the temperature ranges for 4 h to ensure that HSP expression was detectable in gill tissues (Bowen et al., 2020). The temperatures for the treatment groups did not overlap with holding aquarium temperatures. Therefore, control groups were sampled at temperatures equivalent to the aquariums (i.e., 16.8°C–19.7°C; *N* = 10). Before euthanasia, we recorded lengths to the nearest 1.0 mm and weights to the nearest 0.1 g of each fish; condition was calculated using Fulton's condition factor (K) (Ricker, 1975). We performed an analysis of variance (ANOVA) post hoc test on length and weights of fish to test for differences among treatment groups and controls. No differences were detected (length: *p* = 0.52; weight: *p* = 0.57). We extracted gill tissue from all euthanized fish and stored them in a preservation buffer, RNAlater (ThermoFisher Scientific, Waltham, MA, USA). We incubated the gill tissue in the buffer overnight at 4°C before storage at −80°C.

**TABLE 1:** Experimental means and standard deviations (SD) of heating rates and trial temperatures for each temperature treatment group of *Ericymba dorsalis* during heat shock protein experiments.

Temperature treatment groups	Sample size	Mean heating rate (°C/min)	SD heating rate (°C/min)	Mean trial temperature (°C)	SD trial temperature (°C)
Control	10	NA	NA	17.9	0.8186
Low (21°C–23°C)	11	0.0602	0.00405	22.2	0.1174
Moderate (25°C–27°C)	10	0.0583	0.00396	26.1	0.0739
High (29°C–31°C)	16	0.0564	0.00629	30.1	0.0945

### 2.4 RNA extraction and cDNA synthesis

We extracted total RNA from gill tissue samples with the MasterPure complete DNA and RNA Purification Kit (LGC Biosearch Technologies, Hoddesdon, UK) according to manufacturer's instructions. The contaminating DNA was removed by adding 200 μL of DNase solution to each sample. We then used RNA Clean and Concentrator‐25 protocol (Zymo Research, Tustin, CA, USA) to further remove impurities and contaminating DNA from the RNA extractions. We quantified our samples to ensure successful RNA extraction and elimination of DNA using Quant‐iT RNA and Quant‐iT dsDNA broad range kits, respectively (ThermoFisher Scientific). Total RNA extractions were stored at −80°C until further processing. A standard complementary DNA (cDNA) synthesis was performed on 1 μg of RNA template from each sample. cDNA synthesis was performed using QuantiTect Reverse Transcription Kit (Qiagen, Redwood, CA, USA). The cDNA samples were stored at −20°C until analysis.

### 2.5 Primer design

We selected two reference genes, tyrosine 3‐monooxygenase/tryptophan 5‐monooxygenase activation protein zeta (YWHAZ) and ribosomal protein L8 (rpL8), and the target gene, heat shock protein family A member 1A (HSP70), for our analysis. We designed primers for YWHAZ, rpL8 and HSP70 based on alignments from several species in the same family (Leuciscidae) as *E. dorsalis* using sequences available at NCBI (www.ncbi.nlm.nih.gov). We designed primers to amplify a ~300‐base pair sequence corresponding to HSP70, YWHAZ and rpL8 (Table 2). Two *E. dorsalis* samples were amplified at the three genes with an Advantage 2 PCR kit (Takara Bio, San Jose, CA, USA). PCR reactions were carried out in 25‐μL volume with 1‐μL cDNA, 1× Advantage 2 buffer, 0.2 mM dNTPs, 0.2 mM of each primer and 1× Advantage 2 polymerase mix. We performed a gradient thermocycler profile that began at 95°C for 2 min followed by 40 cycles of 95°C for 30 s, 56°C–70°C for 30 s, 68°C for 1 min and concluded with 68°C for 1 min. We visualized PCR products using gel electrophoresis (1.5% agarose gel stained with SyberGreen I; ThermoFisher Scientific). Excess dNTPs and primers were removed from PCR reactions with ExoSap‐IT (ThermoFisher Scientific), and PCR products were cycle‐sequenced at Functional BioSciences (Madison, WI, USA). Forward and reverse sequences were reconciled using Sequencher (Gene Codes Corporation, Ann Arbor, MI, USA). Based on the *E. dorsalis* sequence data, we designed species‐specific primers targeting ~100‐base pair gene segments for subsequent quantitative PCR analysis (Table 2). Successful amplification of target genes was confirmed for species‐specific primer efficacy by cycle‐sequencing, as described above. Sequences are accessioned in NCBI GenBank (pending).

**TABLE 2:** Nucleotide sequences of primers designed to amplify two reference genes, tyrosine 3‐monooxygenase/tryptophan 5‐monooxygenase activation protein zeta (YWHAZ), and ribosomal protein L8 (rpL8) and the target gene heat shock protein family A member 1A (HSP70) for *Ericymba dorsalis*.

Gene	Primer name	Primer efficiency	Primer sequence (5′‐3′)
HSP70	HSP70_402F		GACCCAGTTGTGCAGTCTGACATG
HSP70	HSP70_ 666R		GGCAATGGCTGCAGCTGTGGG
YWHAZ	YWHAZ_243F		CCTAGCAGCCATGGACAAGAGCC
YWHAZ	YMHAZ_540R		GCTGGAGAAGTGCTGCGGATCAG
rpL8	rpL8_272F		CAAAGCCCATGTCAAGCACAG
rpL8	rpL8_602R		GGGTTATGGGAGATGACTGTGGCG
HSP70	BMS_HSP70_526F	106.6%	GGTCCTGGTGAAGATGAAGG
HSP70	BMS_HSP70_625R		TTGAGCCCAGCGATCACTCC
YWHAZ	BMS_YWHAZ_288F	107.6%	AGCAGGCCGAACGCTACG
YWHAZ	BMS_YWHAZ_389R		CCAGGCGGACCGGCGAGCG
rpL8	BMS_rpL8_476F	103.8%	GGGCAGTTCATCTACTGTGGC
rpL8	BMS_rpL8_564R		GCAAGCTTGCCCCGGTCTCC

*Note*: Primer names with ‘BMS’ denote species‐specific primer designs used for the quantitative polymerase chain reaction (PCR), and primer efficiencies are listed.

### 2.6 Estimation of heat shock protein mRNA expression

The species‐specific primers were used for the quantitative PCR (qPCR) analysis. Primer efficiency was comparable across loci as estimated from a standard curve on serial dilution series (Table 2). The cDNA products were used as templates for amplifying the reference (YWHAZ and rpL8) and target (HSP70) genes. We used Bio‐Rad SsoAdvanced Universal SYBR Green Supermix Taq (Bio‐Rad, Hercules, CA, USA) following manufacturer protocol. Reactions were carried out in 10‐μL volumes with 1‐μL cDNA, 1× Sso Advance Mix and 0.2 mM of each primer. Thermocycler profiles began at 95°C for 30 s followed by 40 cycles of 95°C for 10 s and 60°C for 20 s. The melt curve analysis started at 65°C for 5 s and 0.5°C increment temperature increases to 95°C. We amplified individuals using a duplicate structure for each gene, and a melting curve analysis was performed after each run to verify the amplification specificity on a BioRad CFX 96. Reference genes and the target gene were amplified on the same 96‐well PCR plate for each individual. Replicates with absolute differences in threshold cycles (C_T_, the amplification cycle that allows for detection) of >1 were re‐amplified.

We evaluated the stability of reference genes using the web‐based analysis tool RefFinder (https://www.ciidirsinaloa.com.mx/RefFinder-master/) (Xie et al., 2023). Based on this analysis, both YWHAZ and rpL8 were found to be stable and subsequently used for normalization. For data analysis, we calculated the geometric mean of the C_T_ values from the reference genes YWHAZ and rpL8 to generate a reference threshold crossing for each sample. We averaged the C_T_ values of each sample for the gene of interest, HSP70. We then normalized values (reference gene threshold crossing subtracted from HSP70 threshold crossing). All analyses were performed on normalized C_T_ values for HSP70; the lower the normalized value, the more transcripts were present. A change in the normalized value of 2 is approximately equivalent to a fourfold change in the amount of the transcript.

### 2.7 Data analysis

We calculated the mean CTmax across all individuals (*N* = 13). Further, we built separate generalized linear models (GLMs) to determine if fish length, condition or the heating rate for individual trials affected CTmax.

We constructed a candidate set of GLMs to evaluate the influence of temperature treatment groups (TTGs), fish condition and heating rate on normalized HSP70 mRNA expression values. Specifically, the individual heating rate used in each trial was examined for its influence on normalized HSP70 values. We ranked the competing models using Akaike's information criterion corrected for a small sample size (AICc; Burnham & Anderson, 2002). We conducted an ANOVA to examine the relation between HSP70 mRNA expression and TTGs. We applied a Tukey honest significant difference (HSD) post hoc test to identify significant differences between TTGs. The initial temperature within a TTG was deemed a thermal stress threshold if the TTG exhibited the first instance of HSP upregulation, as determined by Tukey's HSD test results. All analyses were performed using Program R Statistical Software (version 4.5.0; R Core Team, 2023), and all statistical analyses were deemed significant at the *α* = 0.05 level.

## 3 RESULTS

**FIGURE 2:**
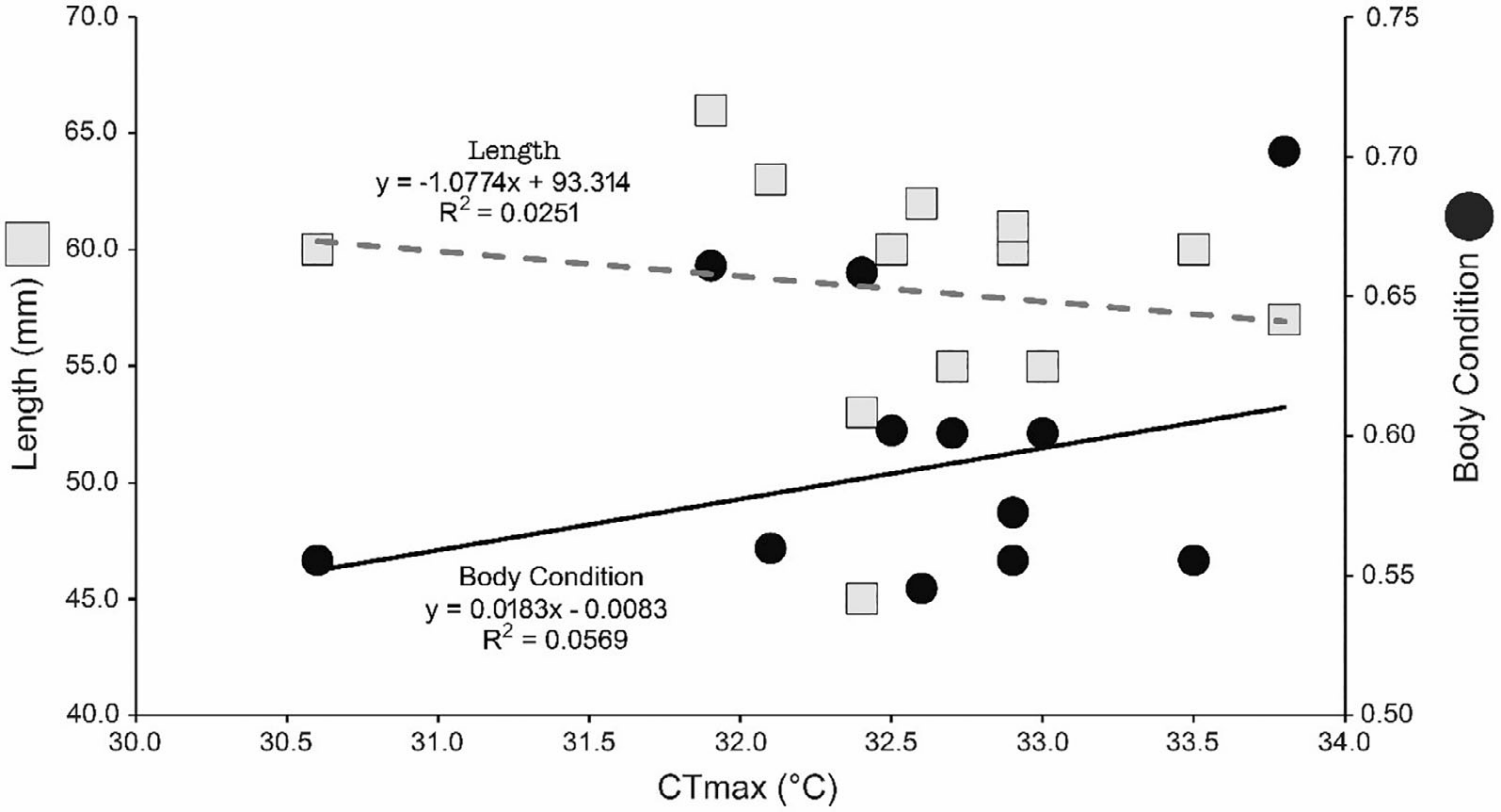
Relationship between body length (mm) and body condition of *Ericymba dorsalis* with critical thermal maximum (CTmax in°C).

The mean CTmax was 32.56°C (SD = 0.78) across all individuals (*N* = 13). We found no significant influence of length, heating rate or body condition on CTmax (*p* > 0.5; Figure 2).

**FIGURE 3:**
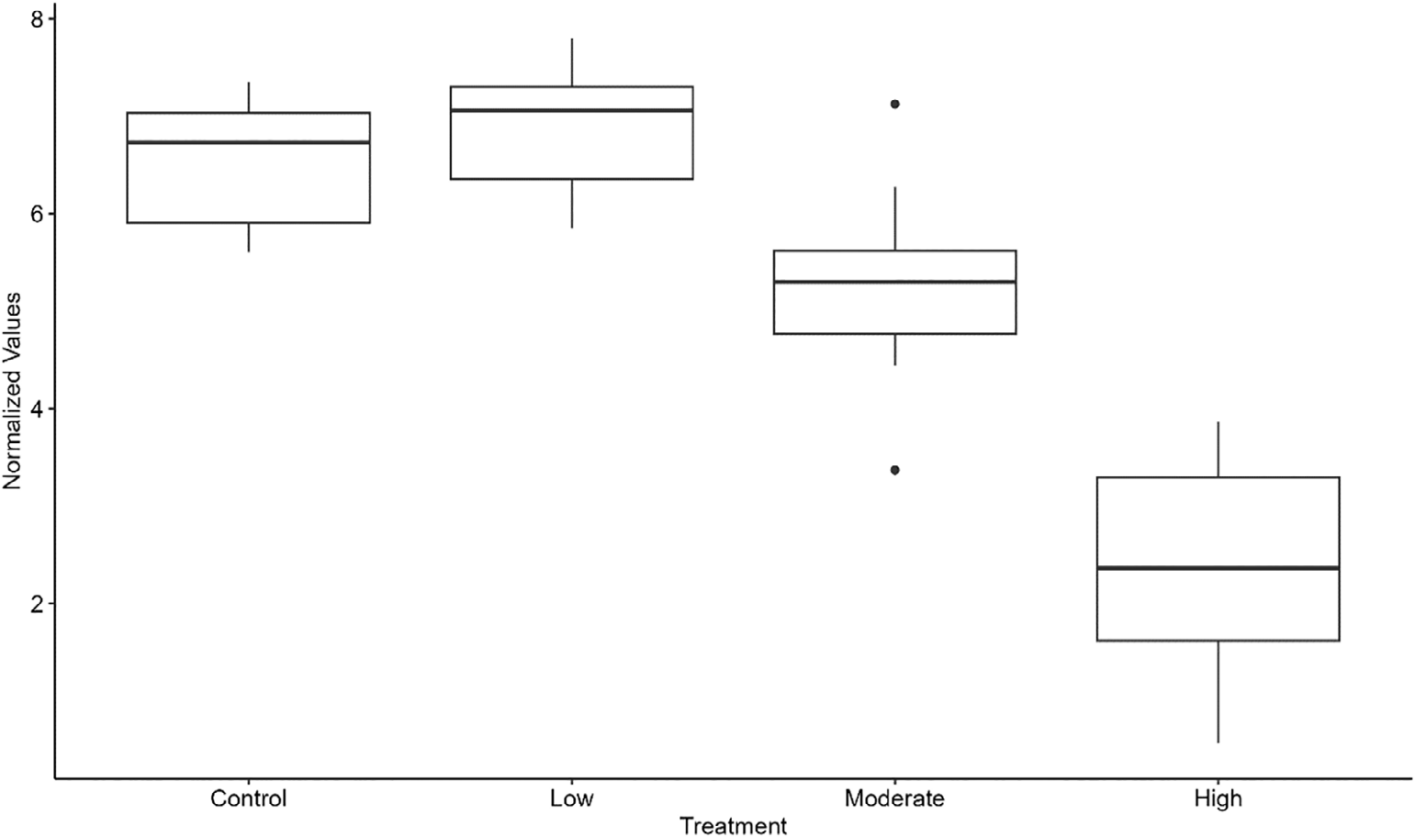
Heat shock protein family A member 1A (HSP70) gene expression among the three water treatment groups (low 21°C–23°C, moderate 25°C–27°C, high 29°C–31°C) of *Ericymba dorsalis*. Low normalized values correlate with HSP70 mRNA upregulation measured using quantitative polymerase chain reaction (qPCR). The bold line represents the median of the data, with the interquartile range (25–75 percentile) denoted by the box.

The top‐ranked model included the HSP70 normalized values and TTGs (Table 3). The TTGs positively influenced HSP70 expression [ANOVA, *F*(3,42) = 71.29, *p* < 2e‐16]. The subsequent Tukey's HSD post hoc test indicated that the moderate and high treatment groups had higher levels of HSP70 expression than the control and low treatment groups. The thermal stress threshold was determined to be 25°C because HSP upregulation was found to be significant in the moderate treatment group, and 25°C was the initial temperature. The low treatment group was not different from the control treatment group (Figure 3).

**TABLE 3:** Model rankings for combined effects between heat shock gene expression, temperature treatment groups of *Ericymba dorsalis* and interactions.

Model	AIC_c_	*Δ*AIC_c_	AIC_c_Wt
HSP70 ~ Treatment	126.55	0.00	0.99
HSP70 ~ Treatment × condition	136.57	10.02	0.01
HSP70 ~ Treatment × heating rate	149.57	23.02	0.00

Abbreviations: AICc, Akaike's information criterion corrected for a small sample size; HSP70, heat shock protein 70.

## 4 DISCUSSION

Our findings demonstrate that *E. dorsalis* possess thermal stress thresholds that are frequently exceeded within Great Plains streams (Figure 1). Future stress events may be exacerbated by the hydrological stochasticity of Great Plains streams. Sudden changes in temperature and flow occur in Great Plains streams due to unpredictable hydrological disturbances (Dodds et al., 2004). Within some streams, groundwater connections to pool habitats may help buffer against abrupt thermal shifts and create areas of thermal refugia for biota (Beatty et al., 2010; Falke et al., 2011; Walters et al., 2024). However, as human demand for water increases, so does the rate of groundwater extraction, thereby lowering water tables and resulting in streams disconnecting from groundwater sources (Hoagstrom et al., 2011; Perkin & Gido, 2011). For instream pool habitats to maintain their groundwater connections and for aquifer recharge to occur, current extraction rates would have to diminish by 75%–80% (Falke et al., 2011; Steward et al., 2013). Furthermore, groundwater extraction, coupled with future climate scenarios, threatens to exacerbate the disturbance regimes within Great Plains streams, potentially leading to a decline in species richness (Cross et al., 1985; Falke et al., 2011; Matthews & Zimmerman, 1990).

A decline in cool‐water refugia could heighten thermal stress affecting *E. dorsalis* and drive range shifts, particularly given predictions that the species' probability of occurrence may decrease by 31%–60% under climate change scenarios (Bouska et al., 2019). Temperature was the primary variable driving range loss; moreover, model projections suggest *E. dorsalis* will be extirpated from most of its current range in Nebraska (Bouska et al., 2019). Nebraska's Sandhills streams provide cool water refugia for fish species because streams in the region are fed by the Ogallala Aquifer, which may dampen large variations in stream temperature (Bleed & Flowerday, 1998; Chen et al., 2003). Fish in groundwater‐fed systems may be buffered against large thermal changes and have likely experienced a relatively stable temperature environment throughout their evolutionary history. Therefore, populations may not have sufficient genetic diversity to cope with larger temperature fluctuations. This may at least partially explain the lower CTmax and the differential expression of HSP estimated in this study. Future stream temperatures projected under various scenarios of environmental change likely will challenge the effectiveness of Sandhills streams to provide cool water refugia due to the shallow groundwater depth, which makes the region more susceptible to warming trends (Hare et al., 2021). In our study, fish started to experience thermal stress around 25°C, signified by the upregulation of HSP70. *E. dorsalis* found in other locations have been observed in conditions that frequently exceed the CTmax derived from this study (Fessell, 1996). Therefore, thermal history of a population likely influences the ability of this species and others to cope (refer to Olsen et al., 2021) with sudden thermal changes.

Our study estimated a lower mean CTmax (32.56°C) of *E. dorsalis* than previous studies (Fessell, 1996; Smale & Rabeni, 1995). We used a heating rate of 0.3°C/min, which is commonly used in CTmax studies. There are concerns, however, that the heating rate we applied may overestimate a species' true thermal limit due to the lag time associated with rapid heating, where the rising temperature may outpace the momentary physiological response of the fish (Desforges et al., 2023). Per results from Fessell (1996) and Smale and Rabeni (1995), we hypothesize that the collection location of wild fish may play a larger role in describing the CTmax of *E. dorsalis* than the heating rate. Fessell (1996) used a heating rate of 1°C/min, and fish attained a mean CTmax of 36.6°C. The lag time between the rising water temperature and the fish's internal temperature may explain the higher estimated CTmax reported by Fessell (1996). In contrast, Smale and Rabeni (1995) also attained a CTmax of 36.6°C, but fish were warmed by 0.016°C/min to mimic the natural warming rate from the wild collection site. The slower heating rate would ensure that fish did not experience a thermal lag, influencing their CTmax. Our study employed ideal CTmax methodology as the summation of previous CTmax studies has suggested that CTmax protocols should be adjusted to account for species‐specific life‐history traits, and using wild‐collected animals yields more ecologically significant results than the use of laboratory‐raised specimens (Ruthsatz et al., 2024). The heating rate we applied is intermediate between the two studies, yet the estimated CTmax is 4.1°C lower than previous studies, suggesting that phenotypic plasticity among collection locations may influence CTmax estimates. Phenotypic plasticity can occur at the population or individual level. The genomes of individual fish were not sequenced across the aforementioned studies; therefore, we do not have evidence to support individual‐level plasticity. However, population‐level plasticity is likely occurring, where multiple alleles for a given gene exist in the population, and each allele expresses a different phenotype. Phenotypic plasticity has been demonstrated in laboratory studies where acclimating fish to higher or lower temperatures can result in different thermal tolerances (Fangue et al., 2006; Morrison et al., 2020). Therefore, it is plausible that *E. dorsalis* have acclimated to their specific (localized) habitat. In this study, fish were collected from the South Fork of the Elkhorn River in Nebraska. This river has substantial groundwater inputs that may stabilize its thermal regime (Bleed & Flowerday, 1998). Consequently, the fish may not be adapted to abnormally high temperatures as reflected by their lower CTmax. In contrast, Fessell (1996) sampled fish from the Platte River in Nebraska, which can exceed 35°C in the summer. *E. dorsalis* in the Platte River may be adapted to withstand higher temperatures, as shown by their elevated CTmax. Similarly, fish collected by Smale and Rabeni (1995) from the Salt River in Missouri likely experience high summer temperatures explaining the higher estimated CTmax. The Salt River has low stream bed permeability and limited groundwater inputs, resulting in highly variable flows after rain events and frequent low‐flow conditions (Dames & Todd, 2003). The upregulation of HSPs in *E. dorsalis* may serve as an adaptive mechanism, increasing their CTmax and broadening thermal tolerance. For example, other studies have observed that lizards adapted to thermally unstable environments utilize constant HSP upregulation to cope with frequent thermal shifts (Ulmasov et al., 1992). Constant HSP upregulation ensures that there is no lag between the creation of the proteins and when they are needed during a thermal stress event. Given the stochastic nature of the Platte and Salt Rivers, *E. dorsalis* may exploit a similar HSP strategy to cope with frequent thermal stress.

Often, model predictions for range expansion and contraction cannot account for the adaptive capacity of populations to environmental changes. Data limitations encompassing the adaptive capacities of small‐bodied non‐game stream fish make model predictions challenging. To mitigate this, future research could develop thermal performance curves (TPCs) for populations that encapsulate the entirety of an organism's thermal tolerance, thereby providing a more robust baseline of an organism's physiological limits (Huey & Stevenson, 1979; Schulte et al., 2011). Additionally, the TPCs of populations may be manipulated through thermal acclimation procedures to test adaptive capacities to environmental changes, including warming temperatures. Incorporating TPCs into mechanistic distribution models may provide more accurate predictions regarding a species' adaptive capacity to changing environmental conditions and subsequent range shifts (Angert et al., 2011). Such models may enable predictions regarding whether the Sandhills ecoregion may provide thermal refugia for species if climatic conditions continue to change.

## 5 CONCLUSION

Thoroughly evaluating a species' thermal tolerance is integral for projecting shifts in distribution or persistence on the landscape. Future research utilizing CTmax experimentation may benefit from standardizing heating rates within a species to simplify the comparison process across studies. By coupling thermal maxima experimental results with stream temperature models, we could support management strategies to preserve or restore thermal refugia. Our study quantified a thermal stress threshold and critical thermal maxima for *E. dorsalis* and found the CTmax of this species to vary across studies. This may suggest that populations of *E. dorsalis* are thermally adapted to their current or past thermal regimes and resiliency, and persistence is likely population dependent and not species wide. This has implications for conservation strategies that may have to assess thermal conditions on a local level rather than making average species assessments. However, in future climate scenarios, thermal adaptation alone may not ensure species' survival, potentially leading to a decline in regional species richness.

## AUTHOR CONTRIBUTIONS

All authors participated in formal analysis, validation and writing – review and editing. Conceptualization by Ella K. Humphrey, Jonathan J. Spurgeon, Lizbeth Bowen and Sarah A. Sonsthagen; data curation by Ella K. Humphrey, Robert E. Wilson and Sarah A. Sonsthagen; investigation by Ella K. Humphrey, Robert E. Wilson, Shannon C. Waters‐Dynes and Sarah A. Sonsthagen; methodology by Ella K. Humphrey, Jonathan J. Spurgeon, Lizbeth Bowen, Robert E. Wilson, Shannon C. Waters‐Dynes and Sarah A. Sonsthagen; project administration and funding by Ella K. Humphrey, Jonathan J. Spurgeon and Sarah A. Sonsthagen; resources by Ella K. Humphrey, Jonathan J. Spurgeon, Braxton M. Newkirk and Sarah A. Sonsthagen; software by the R Core Team; supervision by Jonathan J. Spurgeon, Robert E. Wilson and Sarah A. Sonsthagen; visualization by Ella K. Humphrey, Jonathan J. Spurgeon and Sarah A. Sonsthagen; writing – original draft by Ella K. Humphrey, Braxton M. Newkirk and Sarah A. Sonsthagen.

## FUNDING INFORMATION

This research was funded by the University of Nebraska‐Lincoln Undergraduate Creative Activities and Research Experience and the Cabela's Apprenticeship Research Program.

## CONFLICT OF INTEREST STATEMENT

The authors declare no conflicts of interest.

## Data Availability

Sequence data are available on NCBI GenBank (accession numbers pending). All data analysed in this study are available upon request from the corresponding author.
